# 2-Amino-*N*′-phenyl­benzohydrazide

**DOI:** 10.1107/S1600536812022362

**Published:** 2012-05-23

**Authors:** Víctor Kesternich, Paulo Gahona, Marcia Pérez-Fehrmann, Iván Brito, Matías López-Rodríguez

**Affiliations:** a Departamento de Química, Universidad Católica del Norte, Casilla 1280, Antofagasta, Chile; b Departamento de Química, Facultad de Ciencias Básicas, Universidad de Antofagasta, Casilla 170, Antofagasta, Chile; c Instituto de Bio-Orgánica ’Antonio González’, Universidad de La Laguna, Astrofísico Francisco Sánchez N°2, La Laguna, Tenerife, Spain

## Abstract

In the title compound, C_13_H_13_N_3_O, the NNCO unit forms dihedral angles of 35.8 (1) and 84.0 (1)° with the benzene and phenyl rings, respectively. The dihedral angles between the aromatic rings is 61.2 (1)°. An intra­molecular N—H⋯O hydrogen bond occurs. In the crystal, mol­ecules are linked by weak N—H⋯O hydrogen bonds into *C*(4) chains parallel to the *c* axis. Neighbouring chains are linked by weak N—H⋯N hydrogen bonds, forming *R* ^4^_4_(20) rings, and resulting in the formation of a two-dimensional network lying parallel to (010). The packing also features π–π stacking inter­actions between phenyl rings [centroid–centroid distance = 3.803 (2) Å].

## Related literature

For the pharmacological activity of quinazolinones, see: Kamal *et al.* (2010 ▶) and of benzotriazepinones, see: Filippakopoulos *et al.* (2012 ▶); Spencer *et al.* (2008 ▶). For the synthesis of the starting material 1*H*-benzo[*d*][1,3]oxazine-2,4-dione, see: Iwakura *et al.* (1976 ▶); Leiby & Heindel (1976 ▶). For hydrogen-bond motifs, see: Bernstein *et al.* (1995 ▶).
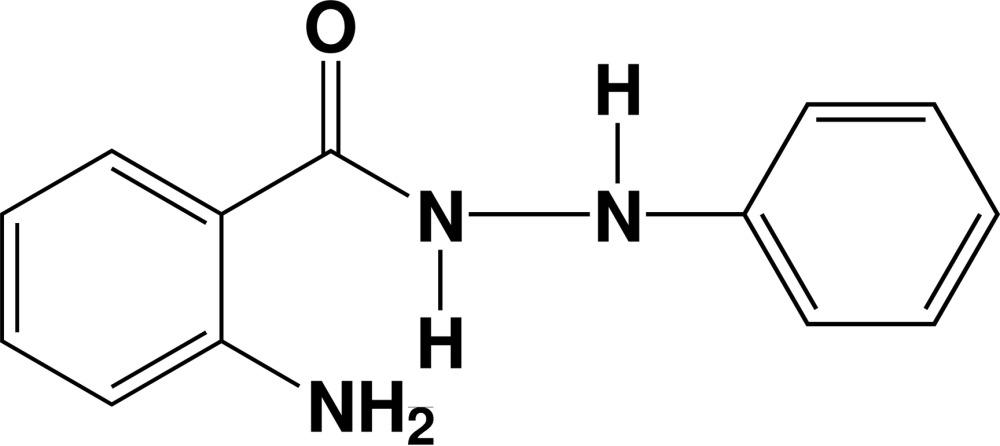


## Experimental

### 

#### Crystal data

C_13_H_13_N_3_O
*M* *_r_* = 227.26
Monoclinic, 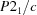

*a* = 6.1190 (12) Å
*b* = 19.921 (4) Å
*c* = 9.6490 (19) Å
β = 94.08 (3)°
*V* = 1173.2 (4) Å^3^
*Z* = 4
Mo *K*α radiation
μ = 0.09 mm^−1^
*T* = 293 K
0.40 × 0.21 × 0.10 mm

#### Data collection

Nonius KappaCCD area-detector diffractometer
17096 measured reflections
2923 independent reflections
2330 reflections with *I* > 2σ(*I*)
*R*_int_ = 0.092

#### Refinement

*R*[*F* ^2^ > 2σ(*F* ^2^)] = 0.068
*wR*(*F* ^2^) = 0.163
*S* = 1.09
2923 reflections
162 parameters
H atoms treated by a mixture of independent and constrained refinement
Δρ_max_ = 0.53 e Å^−3^
Δρ_min_ = −0.49 e Å^−3^

### 

Data collection: *COLLECT* (Nonius, 2000 ▶); cell refinement: *DENZO-SMN* (Otwinowski & Minor, 1997 ▶); data reduction: *DENZO-SMN*; program(s) used to solve structure: *SHELXS97* (Sheldrick, 2008 ▶); program(s) used to refine structure: *SHELXL97* (Sheldrick, 2008 ▶); molecular graphics: *OLEX2* (Dolomanov *et al.*, 2009 ▶); software used to prepare material for publication: *WinGX* (Farrugia, 1999 ▶) and *publCIF* (Westrip, 2010 ▶).

## Supplementary Material

Crystal structure: contains datablock(s) I, global. DOI: 10.1107/S1600536812022362/ds2196sup1.cif

Structure factors: contains datablock(s) I. DOI: 10.1107/S1600536812022362/ds2196Isup2.hkl

Supplementary material file. DOI: 10.1107/S1600536812022362/ds2196Isup3.cml

Additional supplementary materials:  crystallographic information; 3D view; checkCIF report

## Figures and Tables

**Table 1:** Hydrogen-bond geometry (Å, °)

*D*—H⋯*A*	*D*—H	H⋯*A*	*D*⋯*A*	*D*—H⋯*A*
N1—H1⋯O1^i^	0.86	2.07	2.903 (2)	162
N3—H3*A*⋯O1	0.86 (3)	2.21 (3)	2.845 (2)	131 (2)
N2—H2⋯N3^ii^	0.86	2.54	3.126 (3)	126

Symmetry codes: (i) 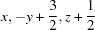; (ii) 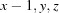.

**Fig. 1.**
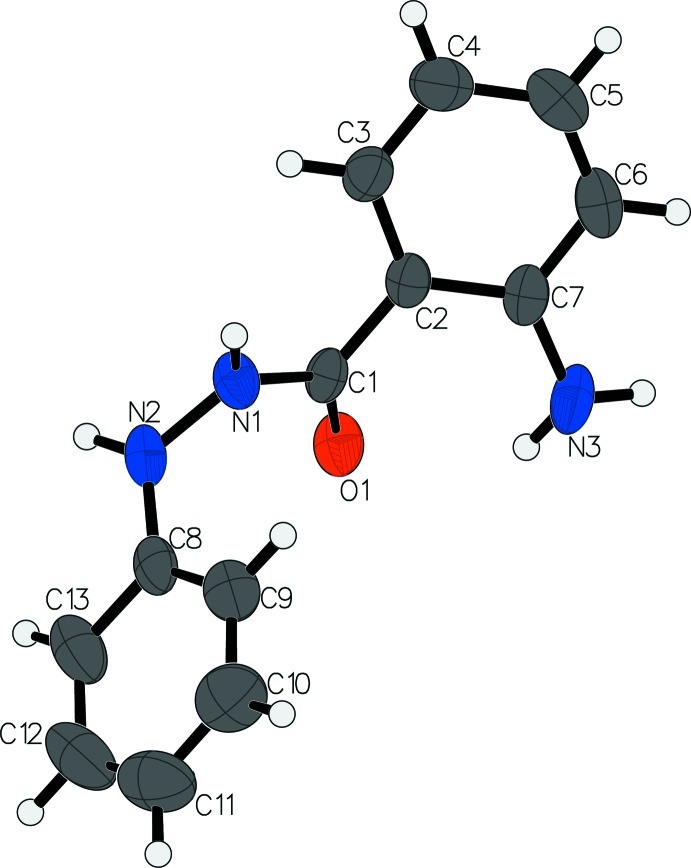
A view of the molecular structure of the title molecule, with the atom numbering. Displacement ellipsoids are drawn at the 50% probability level.

**Fig. 2.**
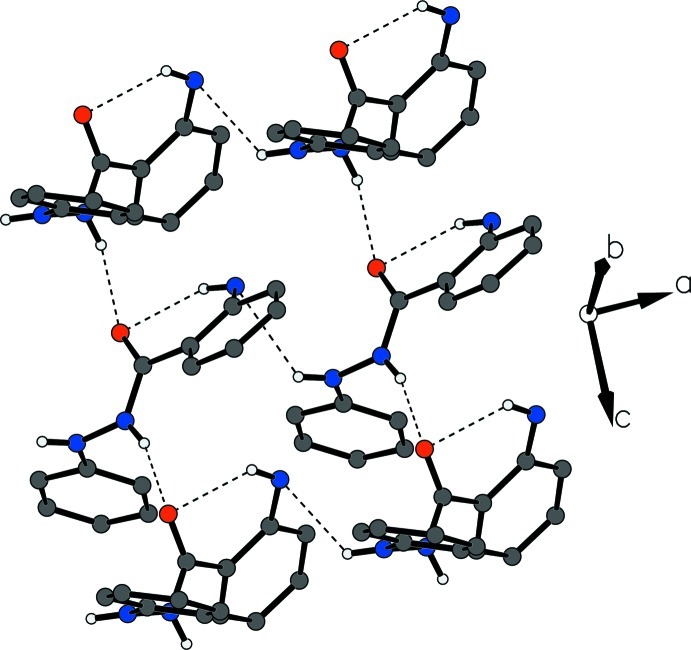
A partial view along the c axis of the crystal packing of the title compound, showing the formation of the N—H··· O hydrogen bonded chain and the centrosymmetric R44 (20) rings [see Table 1 for details; the H-atoms not involved in hydrogen-bonding have been omitted for clarity]

## supplementary crystallographic information

### Comment

The 2-amino-*N*'-phenylbenzohydrazide 2 is a key intermediate to obtain quinazolinones and benzotriazepines derivatives. The quinazolinone nucleous and its derivatives have been extensively studied because of their wide range of pharmacological activities, including antiviral, antibacterial, antifungal, antimalarial, anticancer, antihypertensive, diuretic, anticonvulsant and anti-inflammatory (Kamal *et al.*, 2010). On the other hand, the benzotriazepinones have been described as efficient enzymatic inhibitors (Filippakopoulos *et al.*, 2012; Spencer *et al.*, 2008). We report herein on the synthesis and crystal structure of the title compound, a member of this important family of compounds. In the title molecule, Fig. 1, the NNCO moiety form a dihedral angle of 35.8 (1)° and 84.0 (1)° with benzene and phenyl rings respectively. The dihedral angles between the aromatic rings is 61.2 (1)°. In the crystal the molecules are packed *via*π–π stacking interaction [centroid–centroid distance 3.803 (2) Å] and linked by N1—H1···O1(*x*, -*y* + 3/2, *z* + 1/2) weak hydrogen bond to form a C(4) chain running parallel to the *c* axis, which are linked to neighboring chains by N2—H3···N3(*x* - 1, *y*, *z)* weak hydrogen bond to form *R*^4^_4_(20) centrosymmetric rings (Bernstein *et al.*, 1995). One intramolecular N—H···O hydrogen bond is observed too, Fig.2, Table1.

### Experimental

The synthesis of the 2-amino-*N*'-phenylbenzohydrazide 2 was done starting of isatoic anhydride (1*H*-benzo[*d*][1,3]oxazine-2,4-dione) (Leiby & Heindel 1976; Iwakura *et al.*, 1976), which was treated with phenyl hydrazine in DMF at reflux by 2 h to give an 82% yield. The product was crystallized in ethyl acetate with melting point 227–228 °C. UV λ(MeOH) 310, 280 and 225 nm, λ(MeONa) 340, 265 and 225 nm. IR cm^-1^, (Nujol), 3300 (NH), 1670 (carbonyl).

### Refinement

The H-atoms could be located in difference Fourier maps. H3A and H3B atoms parameters were freely refined. The remaining H atoms, were positioned geometrically and treated using a riding model with N—H = 0.86 Å, C—H = 0.93 with *U*_iso_(H) = k × *U*_eq_(N,*C*), where k = 1.2 for both atoms.

### Crystal data

**Table:**

C_13_H_13_N_3_O	*F*(000) = 480
*M**_r_* = 227.26	*D*_x_ = 1.287 Mg m^−^^3^
Monoclinic, *P*2_1_/*c*	Mo *K*α radiation, λ = 0.71073 Å
Hall symbol: -P 2ybc	Cell parameters from 2923 reflections
*a* = 6.1190 (12) Å	θ = 3.9–28.6°
*b* = 19.921 (4) Å	µ = 0.09 mm^−^^1^
*c* = 9.6490 (19) Å	*T* = 293 K
β = 94.08 (3)°	Block, colourless
*V* = 1173.2 (4) Å^3^	0.40 × 0.21 × 0.10 mm
*Z* = 4	

### Data collection

**Table:**

Nonius KappaCCD area-detector diffractometer	2330 reflections with *I* > 2σ(*I*)
Radiation source: fine-focus sealed tube	*R*_int_ = 0.092
Graphite monochromator	θ_max_ = 28.6°, θ_min_ = 3.9°
φ and ω scans with κ offsets	*h* = 0→8
17096 measured reflections	*k* = 0→26
2923 independent reflections	*l* = −12→12

### Refinement

**Table:**

Refinement on *F*^2^	Primary atom site location: structure-invariant direct methods
Least-squares matrix: full	Secondary atom site location: difference Fourier map
*R*[*F*^2^ > 2σ(*F*^2^)] = 0.068	Hydrogen site location: inferred from neighbouring sites
*wR*(*F*^2^) = 0.163	H atoms treated by a mixture of independent and constrained refinement
*S* = 1.09	*w* = 1/[σ^2^(*F*_o_^2^) + (0.0568*P*)^2^ + 0.7268*P*] where *P* = (*F*_o_^2^ + 2*F*_c_^2^)/3
2923 reflections	(Δ/σ)_max_ < 0.001
162 parameters	Δρ_max_ = 0.53 e Å^−^^3^
0 restraints	Δρ_min_ = −0.49 e Å^−^^3^

### Special details

**Table:**

Geometry. All s.u.'s (except the s.u. in the dihedral angle between two l.s. planes) are estimated using the full covariance matrix. The cell s.u.'s are taken into account individually in the estimation of s.u.'s in distances, angles and torsion angles; correlations between s.u.'s in cell parameters are only used when they are defined by crystal symmetry. An approximate (isotropic) treatment of cell s.u.'s is used for estimating s.u.'s involving l.s. planes.
Refinement. Refinement of *F*^2^ against ALL reflections. The weighted *R*-factor *wR* and goodness of fit *S* are based on *F*^2^, conventional *R*-factors *R* are based on *F*, with *F* set to zero for negative *F*^2^. The threshold expression of *F*^2^ > 2σ(*F*^2^) is used only for calculating *R*-factors(gt) *etc*. and is not relevant to the choice of reflections for refinement. *R*-factors based on *F*^2^ are statistically about twice as large as those based on *F*, and *R*- factors based on ALL data will be even larger.

### Fractional atomic coordinates and isotropic or equivalent isotropic displacement parameters (Å^2^)

**Table:**

	*x*	*y*	*z*	*U*_iso_*/*U*_eq_	
O1	0.6118 (2)	0.71063 (8)	0.36635 (13)	0.0430 (4)	
N1	0.5506 (3)	0.73222 (8)	0.58950 (15)	0.0377 (4)	
H1	0.5943	0.7527	0.6648	0.045*	
N2	0.3725 (3)	0.68841 (9)	0.59083 (17)	0.0407 (4)	
H2	0.2401	0.7021	0.5736	0.049*	
N3	1.0637 (3)	0.73656 (12)	0.3383 (2)	0.0499 (5)	
H3A	0.956 (5)	0.7108 (13)	0.312 (3)	0.059 (8)*	
H3B	1.161 (5)	0.7445 (14)	0.273 (3)	0.073 (8)*	
C1	0.6555 (3)	0.74322 (9)	0.47424 (17)	0.0313 (4)	
C2	0.8220 (3)	0.79772 (9)	0.48509 (17)	0.0323 (4)	
C3	0.7862 (3)	0.85526 (10)	0.5633 (2)	0.0421 (5)	
H3	0.6585	0.8587	0.6095	0.051*	
C4	0.9359 (4)	0.90690 (12)	0.5732 (3)	0.0560 (6)	
H4	0.9095	0.9450	0.6253	0.067*	
C5	1.1263 (4)	0.90142 (13)	0.5046 (3)	0.0575 (6)	
H5	1.2291	0.9359	0.5115	0.069*	
C6	1.1644 (3)	0.84588 (12)	0.4269 (2)	0.0483 (5)	
H6	1.2936	0.8431	0.3820	0.058*	
C7	1.0137 (3)	0.79316 (10)	0.41334 (18)	0.0364 (4)	
C8	0.4207 (3)	0.62058 (11)	0.62176 (19)	0.0387 (4)	
C9	0.6191 (4)	0.59962 (12)	0.6849 (2)	0.0484 (5)	
H9	0.7320	0.6304	0.7030	0.058*	
C10	0.6494 (5)	0.53310 (14)	0.7208 (3)	0.0668 (7)	
H10	0.7826	0.5195	0.7642	0.080*	
C11	0.4861 (6)	0.48666 (14)	0.6935 (3)	0.0756 (9)	
H11	0.5089	0.4417	0.7165	0.091*	
C12	0.2896 (6)	0.50745 (15)	0.6322 (3)	0.0744 (9)	
H12	0.1780	0.4763	0.6141	0.089*	
C13	0.2535 (4)	0.57384 (14)	0.5967 (2)	0.0574 (6)	
H13	0.1181	0.5873	0.5562	0.069*	

### Atomic displacement parameters (Å^2^)

**Table:**

	*U*^11^	*U*^22^	*U*^33^	*U*^12^	*U*^13^	*U*^23^
O1	0.0418 (8)	0.0618 (9)	0.0262 (6)	−0.0055 (7)	0.0065 (5)	−0.0045 (6)
N1	0.0373 (8)	0.0497 (10)	0.0269 (7)	−0.0088 (7)	0.0078 (6)	−0.0030 (6)
N2	0.0271 (8)	0.0565 (11)	0.0392 (9)	−0.0040 (7)	0.0075 (6)	0.0005 (7)
N3	0.0307 (9)	0.0768 (14)	0.0433 (10)	0.0021 (9)	0.0102 (8)	−0.0160 (9)
C1	0.0273 (8)	0.0423 (10)	0.0244 (8)	0.0062 (7)	0.0036 (6)	0.0025 (7)
C2	0.0314 (9)	0.0411 (10)	0.0247 (8)	0.0037 (7)	0.0042 (6)	0.0069 (7)
C3	0.0428 (11)	0.0431 (11)	0.0416 (11)	0.0052 (9)	0.0110 (8)	0.0008 (8)
C4	0.0652 (15)	0.0420 (12)	0.0619 (14)	−0.0052 (11)	0.0122 (12)	−0.0036 (10)
C5	0.0571 (14)	0.0535 (14)	0.0623 (15)	−0.0169 (11)	0.0069 (11)	0.0088 (11)
C6	0.0358 (10)	0.0681 (15)	0.0418 (11)	−0.0061 (10)	0.0080 (8)	0.0098 (10)
C7	0.0312 (9)	0.0518 (11)	0.0264 (8)	0.0050 (8)	0.0029 (7)	0.0054 (7)
C8	0.0369 (10)	0.0527 (12)	0.0277 (9)	−0.0090 (9)	0.0110 (7)	−0.0053 (8)
C9	0.0466 (12)	0.0556 (13)	0.0429 (11)	−0.0047 (10)	0.0032 (9)	−0.0023 (9)
C10	0.0792 (18)	0.0638 (17)	0.0579 (15)	0.0090 (14)	0.0075 (13)	0.0107 (12)
C11	0.115 (3)	0.0522 (16)	0.0625 (17)	−0.0101 (17)	0.0263 (17)	0.0061 (12)
C12	0.094 (2)	0.0721 (19)	0.0589 (16)	−0.0427 (17)	0.0213 (15)	−0.0099 (13)
C13	0.0503 (13)	0.0728 (17)	0.0496 (13)	−0.0242 (12)	0.0069 (10)	−0.0072 (11)

### Geometric parameters (Å, º)

**Table:**

O1—C1	1.240 (2)	C5—C6	1.366 (4)
N1—C1	1.341 (2)	C5—H5	0.9300
N1—N2	1.397 (2)	C6—C7	1.397 (3)
N1—H1	0.8600	C6—H6	0.9300
N2—C8	1.411 (3)	C8—C9	1.384 (3)
N2—H2	0.8600	C8—C13	1.392 (3)
N3—C7	1.386 (3)	C9—C10	1.379 (4)
N3—H3A	0.86 (3)	C9—H9	0.9300
N3—H3B	0.91 (3)	C10—C11	1.374 (4)
C1—C2	1.487 (3)	C10—H10	0.9300
C2—C3	1.398 (3)	C11—C12	1.366 (5)
C2—C7	1.407 (2)	C11—H11	0.9300
C3—C4	1.376 (3)	C12—C13	1.380 (4)
C3—H3	0.9300	C12—H12	0.9300
C4—C5	1.385 (4)	C13—H13	0.9300
C4—H4	0.9300		
			
C1—N1—N2	121.98 (15)	C5—C6—C7	121.5 (2)
C1—N1—H1	119.0	C5—C6—H6	119.2
N2—N1—H1	119.0	C7—C6—H6	119.2
N1—N2—C8	116.67 (15)	N3—C7—C6	119.45 (18)
N1—N2—H2	121.7	N3—C7—C2	122.17 (19)
C8—N2—H2	121.7	C6—C7—C2	118.23 (18)
C7—N3—H3A	116.4 (18)	C9—C8—C13	119.1 (2)
C7—N3—H3B	113.5 (18)	C9—C8—N2	123.09 (18)
H3A—N3—H3B	115 (3)	C13—C8—N2	117.7 (2)
O1—C1—N1	121.54 (17)	C10—C9—C8	119.9 (2)
O1—C1—C2	123.18 (16)	C10—C9—H9	120.0
N1—C1—C2	115.26 (15)	C8—C9—H9	120.0
C3—C2—C7	119.10 (18)	C11—C10—C9	121.1 (3)
C3—C2—C1	120.27 (16)	C11—C10—H10	119.5
C7—C2—C1	120.60 (17)	C9—C10—H10	119.5
C4—C3—C2	121.46 (19)	C12—C11—C10	119.0 (3)
C4—C3—H3	119.3	C12—C11—H11	120.5
C2—C3—H3	119.3	C10—C11—H11	120.5
C3—C4—C5	119.1 (2)	C11—C12—C13	121.2 (3)
C3—C4—H4	120.5	C11—C12—H12	119.4
C5—C4—H4	120.5	C13—C12—H12	119.4
C6—C5—C4	120.6 (2)	C12—C13—C8	119.7 (3)
C6—C5—H5	119.7	C12—C13—H13	120.2
C4—C5—H5	119.7	C8—C13—H13	120.2
			
C1—N1—N2—C8	89.2 (2)	C3—C2—C7—N3	177.46 (18)
N2—N1—C1—O1	−6.4 (3)	C1—C2—C7—N3	−4.6 (3)
N2—N1—C1—C2	172.28 (16)	C3—C2—C7—C6	1.9 (3)
O1—C1—C2—C3	142.55 (19)	C1—C2—C7—C6	179.85 (16)
N1—C1—C2—C3	−36.1 (2)	N1—N2—C8—C9	17.7 (3)
O1—C1—C2—C7	−35.3 (3)	N1—N2—C8—C13	−167.37 (17)
N1—C1—C2—C7	146.01 (17)	C13—C8—C9—C10	0.6 (3)
C7—C2—C3—C4	−1.0 (3)	N2—C8—C9—C10	175.5 (2)
C1—C2—C3—C4	−178.95 (19)	C8—C9—C10—C11	0.8 (4)
C2—C3—C4—C5	−0.3 (3)	C9—C10—C11—C12	−1.3 (4)
C3—C4—C5—C6	0.7 (4)	C10—C11—C12—C13	0.4 (4)
C4—C5—C6—C7	0.3 (4)	C11—C12—C13—C8	0.9 (4)
C5—C6—C7—N3	−177.3 (2)	C9—C8—C13—C12	−1.4 (3)
C5—C6—C7—C2	−1.6 (3)	N2—C8—C13—C12	−176.6 (2)

### Hydrogen-bond geometry (Å, º)

**Table:**

*D*—H···*A*	*D*—H	H···*A*	*D*···*A*	*D*—H···*A*
N1—H1···O1^i^	0.86	2.07	2.903 (2)	162
N3—H3*A*···O1	0.86 (3)	2.21 (3)	2.845 (2)	131 (2)
N2—H2···N3^ii^	0.86	2.54	3.126 (3)	126

Symmetry codes: (i) *x*, −*y*+3/2, *z*+1/2; (ii) *x*−1, *y*, *z*.
